# The association between interoception and psychopathic traits

**DOI:** 10.3389/fpsyg.2025.1669147

**Published:** 2026-01-07

**Authors:** Yu Gao, Amanda Murphy, Liat Kofler

**Affiliations:** 1 Department of Psychology, Brooklyn College, City University of New York, Brooklyn, NY, United States; 2 The Graduate Center, City University of New York, New York, NY, United States

**Keywords:** autonomic, empathy, interoceptive, psychopathy, psychophysiological

## Abstract

**Introduction:**

Psychopathic traits are characterized by pervasive emotional deficits, including diminished empathy and remorse, heightened impulsivity, and antisocial behavior. Prior work has suggested that these traits may be partly shaped by physiological abnormality, although their associations with interoception—defined as the sensitivity to and awareness of internal bodily signals—has been inconsistent and understudied. The present study aimed to clarify the nature of emotional impairments in psychopathy by examining potential interoceptive abnormalities during a social stress task in a non-incarcerated, non-clinical sample.

**Methods:**

One hundred and fourteen participants (62% women; mean age = 22) from a local college and the surrounding community self-reported psychopathic traits, and their skin conductance level (SCL) was recorded while they experienced social exclusion during a computerized task. Following the task, they reported bodily sensations as an index of interoceptive sensibility. Based on the correspondence between subjective sensations and physiological responses, three interoceptive profiles were identified: an under-reporting group (greater SCL relative to reported sensations), an over-reporting group (lower SCL relative to reported sensations), and a control group (broad match between SCL and reported sensations).

**Results:**

Self-Centered Impulsivity and Coldheartedness were positively and negatively associated, respectively, with interoceptive sensibility. In addition, the over-reporting group scored higher on Self-Centered Impulsivity than the other two groups.

**Discussion:**

Findings suggest that interoceptive disturbances may be particularly characteristic of the impulsive and antisocial behavioral dimension of psychopathy, which is associated with a high tendency to hyper-vigilance and over-report of bodily sensations.

## 1 Introduction

### 1.1 Psychopathic traits

The U.S. maintains one of the highest recidivism rates internationally: 44% of all incarcerated individuals and 39% of young adults return to prison within 1 year of release (Puzzanchera et al., 2022). These persistently elevated rates underscore the importance of identifying developmental, psychological, and neurobiological mechanisms that increase vulnerability to criminal behavior. One of the most robust correlates of criminality, violence, and recidivism is the presence of psychopathic traits, a constellation of affective, interpersonal, and behavioral characteristics marked by callousness, shallow affect, low empathy, impulsivity, and antisocial behavior (Hare, 2003). Classical perspectives emphasized affective deficits and low fear (e.g., Cleckley, 1941; Lykken, 1995), whereas contemporary accounts focus on separable dimensions such as Boldness, Meanness, and Disinhibition (the Triarchic model; Patrick et al., 2009) or on distinct affective–interpersonal versus lifestyle–antisocial components (as operationalized by the Psychopathy Checklist-Revised [PCL-R], Hare, 2003; Skeem and Cauffman, 2003). Although early psychopathy research focused primarily on incarcerated or clinical samples, substantial empirical evidence has established that psychopathic traits are continuously distributed across the general population and detectable in non-forensic community samples (Babiak et al., 2010; Drislane et al., 2014; Lilienfeld et al., 2014; Neumann and Hare, 2008). This dimensional perspective has motivated the development of measurement tools suited for non-clinical populations.

For community and non-forensic samples, self-report frameworks—most notably the Psychopathic Personality Inventory–Revised (PPI-R; Lilienfeld and Widows, 2005)—offer reliable dimensional measurement. The PPI-R aggregates eight subscales, with seven of them loaded onto two higher-order factors—Fearless Dominance and Self-Centered Impulsivity—that map broadly onto the above mentioned affective–interpersonal and lifestyle–antisocial dimensions, respectively. Fearless Dominance includes traits such as social potency, fearlessness, and stress immunity, whereas Self-Centered Impulsivity captures traits such as Machiavellian egocentricity, blame externalizing, carefree nonplanfulness, and impulsive nonconformity (Lilienfeld and Widows, 2005). In addition, the eighth subscale, Coldheartedness, reflects callousness, reduced empathy, and emotional detachment, aligns closely with the affective facets of the PCL-R as well as the Meanness construct (e.g., shallow affect, callous-lack of empathy) in the Triarchic model (which also includes Boldness and Disinhibition; Patrick, 2022). It is largely orthogonal to Fearless Dominance and Self-Centered Impulsivity, which uniquely positions it as a purer indicator of affective poverty (Berg et al., 2015). However, since it includes a limited number of items, shows restricted content coverage (primarily low empathy rather than emotional processing deficits more broadly), and bears attenuated associations with physiological responses (Carré et al., 2013; Contreras-Rodríguez et al., 2014; Decety et al., 2014; Gordon et al., 2004; Seara-Cardoso et al., 2016), its conceptualization and correlates have been understudied (Berg et al., 2015; Marcus et al., 2013; Miller and Lynam, 2012).

### 1.2 Interoception

Interoception denotes the perception, interpretation, and integration of internal bodily signals (e.g., cardiac, respiratory, gastric) that contribute to emotion, decision making, and homeostatic regulation (Craig, 2009). Modern conceptualizations separate interoception into distinct dimensions. Interoceptive accuracy, the objective monitoring and correct identification of bodily sensations as they are occurring, is usually assessed via objective performance on detection tasks, e.g., heartbeat counting/discrimination (Plans et al., 2021; Schandry, 1981). Interoceptive sensibility, the self-perceived sensitivity to internal bodily sensations and the tendency to bring attention or focus to interoceptive signal, is typically measured by self-reported attention to and subjective intensity of bodily sensations (Cabrera et al., 2018; Mehling et al., 2012; Murphy et al., 2020). This distinction is well established in contemporary reviews and methodological papers (Garfinkel et al., 2015; Murphy et al., 2019) and is crucial because objective and subjective indices often diverge and associate differently with emotion and psychopathology (Desmedt et al., 2025). Growing evidence links interoceptive function to emotion regulation, threat appraisal, and social cognition (Desmedt et al., 2023), all processes relevant to psychopathic traits. Further, neuroimaging data has supported that the insular-frontotemporal region, a primary interoception network, also converges with emotional processing and regulation (Adolfi et al., 2017) and has been found to be disrupted in individuals with psychopathic traits (Yang and Raine, 2009).

Empirical research examining the association between psychopathic traits and interoception remains limited. Early work using objective heartbeat detection tasks reported that antisocial or lifestyle facets—particularly those indexing impulsive and antisocial behavior—are associated with reduced interoceptive accuracy (Nentjes et al., 2013). Subsequent community studies have found convergent evidence linking the lifestyle facet of psychopathy to poorer heartbeat detection and reduced self-reported bodily sensibility (Campos et al., 2023; Lyons and Hughes, 2015). Conversely, constructs related to boldness or fearless dominance sometimes show different or even positive relations with certain interoceptive indices, consistent with their low-anxiety profile (Campos et al., 2023). Finally, one study has reported dissociations between physiological reactivity and subjective sensation in individuals high on interpersonal–affective traits, suggesting a mismatch rather than a simple deficit (e.g., reduced autonomic responses paired with inconsistently reported sensations; Gao et al., 2012). Although both interoceptive sensibility (verbal reporting of the magnitude of body sensations) and interoceptive accuracy (comparing verbal report to objective physiological changes) were examined, it was unknown if those with high interpersonal-affective traits over or under reported their bodily sensations. Finally, null findings have emerged in forensic samples (Lamoureux and Glenn, 2021; Zwets et al., 2014). Measurement heterogeneity—differences in tasks, physiological indices (e.g., heart rate vs. skin conductance), and questionnaire scales—complicates synthesis (Desmedt et al., 2023). Overall, evidence tentatively indicates that atypical interoception may be most pronounced for impulsive–antisocial dimensions of psychopathy, but substantive gaps remain.

### 1.3 Current study

The current study attempted to address several research gaps. First, few studies examine objective interoceptive measures using concurrent physiological indices during ecologically valid emotional or social stressors. Although individuals with high psychopathic traits may be able to direct awareness to their body when prompted, they may not naturally be aware of their bodily reactions during emotional or stressful situations (Gao et al., 2012), making it difficult to adapt to their environment appropriately. Reduced awareness of visceral responses—particularly when exposed to aversive tasks such as the Cyberball social exclusion paradigm, may impair adaptive decision-making and contribute to negative outcomes among individuals high in psychopathic traits (Damasio, 1994; Gunschera et al., 2022). Second, little empirical work has tested whether different psychopathy dimensions (Fearless Dominance, Self-Centered Impulsivity, and Coldheartedness) relate to distinct patterns of interoceptive functioning. In particular, Coldheartedness’ association with emotional detachment—but relatively weak association with arousal-based deficits—raises questions about whether these traits are associated with subjective under-reporting, reduced emotional embodiment, or no interoceptive differences at all. Given its conceptual ties to callousness and emotional detachment, one might expect reduced interoceptive awareness or diminished subjective bodily cues during affective states. Third, the directionality of subjective physiological mismatches (over-reporting vs. under-reporting) across psychopathy dimensions is unresolved.

The present study therefore examined interoceptive accuracy and interoceptive sensibility during a validated social stress induction task (Cyberball) and related these indices to Fearless Dominance, Self-Centered Impulsivity, and Coldheartedness in a non-clinical, non-forensic community sample. It was hypothesized that (1) higher Self-Centered Impulsivity would be associated with lower interoceptive accuracy and heightened self-reported bodily sensibility, due to its association with anxiety and emotional dysregulation; and (2) higher Fearless Dominance would be associated with lower interoceptive accuracy and reduced self-reported bodily sensibility, consistent with emotional blunting. Furthermore, because Gao et al. (2012) were unable to determine whether individuals high in psychopathic traits tended to under-report or over-report their bodily sensations, we sought to clarify this issue by delineating three interoceptive profiles: under-reporting group (reduced subjective sensation relative to physiological arousal), over-reporting group (higher subjective sensation relative to physiological arousal), and a control group (broad match between verbal report and objective measure of arousal). We expected that the under-reporting group would score higher on Fearless Dominance than the other two groups, whereas the over-reporting group would have elevated Self-Centered Impulsivity score. Finally, exploratory analyses would probe the role of Coldheartedness due to limited research concerning this facet. Given evidence that psychopathic traits may vary as a function of age and sex (Huchzermeier et al., 2008; Maurer et al., 2022), these two variables were included as covariates in the analyses when they showed significant correlations with the primary variables of interest.

## 2 Methods

### 2.1 Participants

This study was part of a larger project examining psychological, physiological, and behavioral responses to social exclusion. A total of 154 participants were enrolled (61.6% women), ranging in age from 18 to 64 years (*M* = 22.20, S.D. = 5.92). All participants had normal or corrected-to-normal vision and were recruited from a college and surrounding community in a large urban U.S. city. Five participants were removed in analysis due to failing attention checks in the PPI-R (see below) and one participant was removed for > 20% missing responses. Another two were removed due to technical issues concerning psychophysiological data collection. Finally, data from 114 participants (mean age = 22.12, 62% women) were used in the current study. This sample was ethnically diverse (24% African American, 22.3% Caucasian, 21.5% Asian/Pacific Islander, 13.2% Hispanic, 7.4% Multi-Racial, 9.1% Other, and 2.5% undisclosed). One participant identified their gender as non-binary, so their data were excluded from analyses when gender was involved. Power analyses based on original hypotheses using G*Power (Faul et al., 2007) indicated that a sample size of 103 was sufficient to produce significant effects with 80% power and a median effect size for repeated measures ANOVA, Poisson regression, or linear multiple regression (Kofler, 2023). Participants were debriefed at the end of the study, and they received either course credit or monetary compensation. The whole procedure lasted approximately 90 min, and all procedures were approved by the college Institutional Review Board.

### 2.2 Measures

#### 2.2.1 The psychopathic personality inventory-revised

(PPI-R; Lilienfeld and Widows, 2005). This measure was developed to assess psychopathic personality traits in non-forensic, community populations. The PPI-R consists of 154 items rated on a 4-point Likert scale (1 = false, 2 = mostly false, 3 = mostly true, 4 = true). The higher order structure of PPI-R consists of 8 subscales, with 7 of them loaded on two dimensions. The Fearless Dominance dimension includes the following subscales: Social Potency (18 items, e.g., “I feel sure of myself when I’m around other people”), Fearlessness (14 items, e.g., “I agree with the motto, if you are bored with life, risk it”), and Stress Immunity (13 items, e.g., “When I’m in a frightening situation, I can ‘turn off’ my fear almost at will”). Fearless Dominance has been highly correlated with the PCL-R, specifically the Interpersonal-Affective factor, and has been labeled as the “selfish, callous and remorseless use of others” component of the PCL-R (Berardino et al., 2005, p. 824). The Self-Centered Impulsivity dimension has been correlated with the Lifestyle-Antisocial factor of the PCL-R, the “chronically unstable, antisocial and socially deviant lifestyle.” It consists of the following subscales: Machiavellian Egocentricity (20 items, e.g., “If I really want to, I can persuade most people of almost anything”), Blame Externalization (15 items, e.g., “I’m sure some people would be pleased to see my fail in life”), Carefree Nonplanfulness (19 items, e.g., “I like to act first and think later”), and Impulsive Nonconformity (16 items, e.g., “I like to poke fun at established traditions”). Finally, the Coldheartedness subscale includes 16 items and does not load on either factor, therefore is analyzed as a separate construct (Benning et al., 2003). In the current sample, Cronbach’s alphas were 0.89 and 0.86, for the Fearless-Dominance and Self-Centered Impulsivity factor, respectively, and 0.75 for the Coldheartedness subscale.

#### 2.2.2 The Cyberball task

Cyberball is a well replicated social task in which participants partake in a ball-tossing game with two other “virtual participants” (Williams and Jarvis, 2006). This paradigm has been used in social ostracism studies to test for potential group differences between being included or excluded by peers (Williams et al., 2000; Williams and Jarvis, 2006). There are multiple versions of the paradigm. In one version, participants are randomly assigned to either the inclusion or exclusion condition. Those in the inclusion condition receive the ball approximately 33% of the trials throughout the task. In contrast, participants in the exclusion condition receive the ball approximately 33% of the throws during the first 10 passes, while during the remainder of the game the two virtual players pass the ball between themselves, and the participant is excluded from the game. Since our study focused on individual differences to social exclusion, an unbalanced group assignment (*N* = 38 inclusion vs. *N* = 121 exclusion) was used to ensure sufficient power for analyses that were conducted for the exclusion group. An independent samples t-test and chi-squared tests showed that the inclusion and exclusion groups did not differ in age (*t* = −0.29, *p* = 0.770), gender (*χ*^2^(2) = 0.71, *p* = 0.700), or race (*χ*^2^(6) = 6.44, *p* = 0.375). In the current study, Cyberball was used as a social stressor task to induce an emotional reaction in participants; therefore, following analyses only included data from those in the exclusion condition.

The experiment was programmed and run with Experiment Builder software (SR Research). Participants sat approximately 60 cm from a flat 17-inch Dell LCD monitor (60 Hz refresh rate). Participants were instructed to play a virtual ball-tossing game with two other participants in other labs. They were told to press 1 on the response pad to pass the ball to the person who was on the right of the screen and to press 2 to pass the ball to the person on the left. The game included 90 throws and lasted for around 5 min. The virtual participants were represented by standardized photos with assigned names which were displayed on the screen during the game, and a probability simulation directed where the ball was tossed. The photos were taken from the Chicago Faces Database Version 3.0 (Ma et al., 2015). The gender of the two virtual participants was matched to the participant, such that female participants played with two female virtual participants. To control for any race confounds, pictures of one White player and one Black player were used in the game. To increase credibility of the task, after the consent procedure researchers asked to take a photo of the participant so the other players in the game would be able to see who they were playing with.

At the completion of the Cyberball task, participants completed the Needs Threat Scale to measure their fundamental needs that have previously been shown to be affected by social exclusion (Williams, 2009). This information was used as a manipulation check. It consisted of four subscales (belonging, self-esteem, control, and meaningful existence) with 5 items in each subscale rated using a 5-point Likert scale ranging from 1 = “not at all” to 5 = “extremely.” The exclusion group showed significantly lower scores on all four subscales, *t* > 4.28, *p* < 0.001, providing evidence that the experimental manipulation was valid and that the Cyberball paradigm elicited an experience of exclusion in this group.

#### 2.2.3 Body sensation measure

Following a prior study (Gao et al., 2012), after the Cyberball task participants completed a questionnaire in which they were asked, “How much did you experience the following body feelings when playing the game?.” A total of 14 items was included: lump in throat, breathing changes, stomach sensations, feel cold, feel hot, heart pounding, tense muscles, perspiration, goose pimples, facial blushing, jelly legs, hands tremble, voice tremble, and eyes well up with tears. Participants rated each statement on a 5-point Likert scale (1 = not at all, 2 = a little, 3 = sometimes, 4 = often, 5 = very often). This measure was used in the current study to allow direct comparison to findings reported by Gao et al. (2012). A total score was calculated by summing the items, with higher scores indicating more body reactions and higher interoceptive sensibility.

#### 2.2.4 Psychophysiological data acquisition and quantification

Psychophysiological measures were recorded using BIOPAC MP150 (Biopac Systems Inc., Goleta, CA) and measured continually during the rest period (5 min) and during the Cyberball task (approximately 4.5 min). Data was analyzed offline using Acqknowledge 4.2 software (Biopac Systems Inc., Goleta, CA). Skin conductance was recorded using a GSR100C amplifier module with two 6 mm diameter silver/silver chloride electrodes (TSD203) attached to the distal portion of the non-dominant first and second finger. Skin conductance level (SCL) assesses tonic aspects of the arousal response and higher values indicate higher sympathetic responses.

Individual maximum values of SCL were identified during the Cyberball task, in which the individual’s peak SCL throughout the task represents the degree of sympathetic response during the task. Next, an average SCL measure during the 5-min rest period was used to determine baseline measures. A difference score was then calculated between task SCL measures and baseline SCL at rest to determine physiological reactivity. Experimenter and/or equipment error resulted in missing SCL baseline and task data in two participants.

### 2.3 Procedure

Upon arrival, participants were informed about the study procedures and provided informed consent. They completed a battery of questionnaires on papers (including PPI-R and demographic information) and computerized tasks while their psychophysiological responses were recorded. To eliminate any potential order effects, half of the participants completed the questionnaires first, and the other half completed the tasks first. To measure a baseline physiological measure, participants were instructed to sit quietly and remain as still as possible while looking at a fixation cross on the screen for 5 min. At the end of the rest period, a research assistant entered the room to verbally give the Cyberball task instruction. Participants were told that they would be playing a virtual ball tossing game with two other players who were participating in the study in different labs. Next participants completed another task (irrelevant to our current study) before psychophysiological electrodes were taken off and being debriefed.

### 2.4 Data preparation

Six outliers for baseline and task SCL variables were identified (3 S.D. beyond mean) and winsorized prior to analyses. In addition, the Body Sensation Measure and age were highly skewed (skewness > 3). Therefore, natural log transformation was conducted for both variables prior to further analyses. In order to determine interoceptive accuracy, a mismatch score was created by comparing SCL reactivity and verbal report of the Body Sensation Measure score during the task. To do this, both SCL reactivity and body sensation scores were transformed into standardized z-scores. A difference score was then computed by subtracting the standardized body sensation scores from the standardized SCL reactivity scores. Next, three profiles were identified: The under-reporting group had scores 1 standard deviation above the mean (i.e., lower subjective reporting of bodily sensations compared to the measured objective physiological response). The over-reporting group had scores 1 standard deviation below the mean (i.e., higher subjective reporting of bodily sensations compared to the measured physiological responses). Lastly, the control group included all other participants, who were considered to have a general match between their verbal report and SCL reactivity. The over-reporting, under-reporting, and control groups had sample sizes of 19, 21, and 73, respectively.

### 2.5 Data analyses

All analyses were conducted in IBM SPSS 27. First, independent samples t-tests were conducted to test for potential effect of gender on main study variables. Potential effect of age was tested using Pearson correlation analysis. To test for the relationships between psychopathic traits and interoceptive sensibility, a hierarchical linear regression was conducted with covariates entered in the first step and the three psychopathic traits entered in the second step to predict the body sensation score.

Two approaches were used to test if each psychopathic factor was associated with interoceptive accuracy. For the dimensional approach, a hierarchical regression was conducted to predict mismatch score as a continuous measure with age/gender entered in the first step and psychopathic traits entered in the second step. Regarding the grouping approach, an ANOVA was conducted to compare the three groups (over-reporting, under-reporting, and control) on psychopathic traits. Pairwise comparisons with Bonferroni correction were then conducted to probe significant differences. Effect sizes are reported using partial *η*^2^ and Cohen’s *d* (Cohen, 1988).

## 3 Results

### 3.1 Descriptive statistics

Descriptive statistics (before transformation) and Pearson correlation coefficients for the main variables are reported in Table 1. Males scored significantly higher than females on Fearless Dominance, *t*(107.90) = −3.13, *p* = 0.002, *M*_male_ = 106.15, S.D. = 16.15, *M*_female_ = 95.80, S.D. = 19.94, *d* = 0.57, Self-Centered Impulsivity, *t*(111) = −2.00, *p* = 0.048; *M*_male_ = 147.85, S.D. = 21.13, *M*_female_ = 139.71, S.D. = 21.27, *d* = 0.38, and Coldheartedness, *t*(111) = −2.97, *p* = 0.004; *M*_male_ = 31.98, S.D. = 6.93, *M*_female_ = 28.29, S.D. = 6.17, *d* = 0.56. Age (after transformation) was not associated with any of the main variables. Therefore, gender (but not age) was included as a covariate in subsequent analyses. Body Sensation Measure scores (after transformation) were positively and negatively associated with Self-Centered Impulsivity (*r* = 0.36, *p* < 0.001) and Coldheartedness (*r* = −0.20, *p* = 0.035), respectively.

**Table 1:** Pearson correlations and descriptive statistics.

Variables	1	2	3	4	5	6	7
1. Age	1						
2. SCL reactivity	−0.08						
3. BSM	0.01	−0.05					
4. Mismatch	−0.06	0.72^***^	−0.72^***^				
5. Fearless-dominance	−0.10	0.06	−0.02	0.06			
6. Self-Centered Impulsivity	−0.16^a^	−0.09	0.36^***^	−0.31^***^	0.04		
7. Coldheartedness	−0.04	−0.11	−0.21^*^	0.06	0.12	0.08	
Mean	22.22	1.90	19.08	0.00	100.32	142.86	29.76
S.D.	6.17	1.74	7.33	1.45	19.16	21.47	6.69
Range	18–54	−1.35 to 7.80	13–65	−4.83 to 4.30	57–151	98–199	17–56
Skewness	3.08	1.05	3.21	−0.29	0.07	0.10	0.81

^*^p < 0.05, ^***^p < 0.001, ^a^*p* < 0.10; BSM, body sensation measure; SCL, skin conductance level.

### 3.2 Interoceptive sensibility

**Figure 1:**
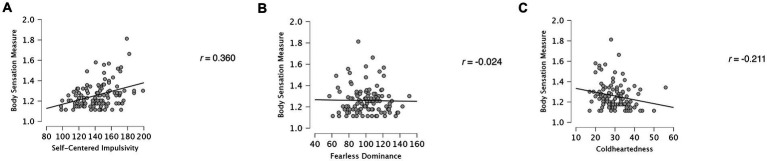
Scatterplots show relationships between body sensation measure with **(A)** Self-Centered Impulsivity, **(B)** Fearless Dominance, and **(C)** Coldheartedness.

In hierarchical linear regressions, gender was entered as a covariate in the first step, and three psychopathic traits variables (Fearless Dominance, Self-Centered Impulsivity, or Coldheartedness) were entered in the second step to predict Body Sensation Measure score. After controlling for gender, adding the three psychopathic variables significantly improved the model (*F* = 5.88, Δ*R*^2^ = 0.178, *p* = <0.001). Although Fearless Dominance did not significantly predict body sensation score (ß = −0.01, *SE* = 0.001, *t* = −0.11, *p* = 0.911), Self-Centered Impulsivity (ß = 0.37, SE = 0.001, *t* = 4.19, *p* < 0.001) and Coldheartedness (ß = −0.23, SE = 0.002, *t* = −2.52, *p* = 0.013) each was significantly associated with body sensation score. Figures 1A–C shows relationships between each psychopathic dimension and Body Sensation Measure scores.

### 3.3 Interoceptive accuracy

#### 3.3.1 Dimensional approach

A hierarchical regression was conducted to predict mismatch score as a continuous measure with gender entered in the first step and psychopathic traits entered in the second step. After controlling for gender, adding three psychopathic variables significantly improved the model (*F* = 3.32, Δ*R^2^* = 0.109, *p* = 0.006). Specifically, Self-Centered Impulsivity significantly and negatively predicted mismatch score (ß = −0.33, SE = 0.006, *t* = −3.54, *p* < 0.001). Effects were not significant for Fearless Dominance (ß = 0.06, SE = 0.007, *t* = 0.68, *p* = 0.558) or Coldheartedness (ß = 0.06, SE = 0.020, *t* = 0.65, *p* = 0.516).

#### 3.3.2 Grouping approach

Given significant gender differences on psychopathic traits, ANCOVAs were run to compare the three groups on psychopathic dimensions with gender as a covariate. Results showed that the three groups differed significantly on Self-Centered Impulsivity, *F* (3,109) = 4.67, *p* = 0.004, partial *η*^2^ = 0.114. Both gender (*F* (1, 109) = 4.57, *p* = 0.035, partial *η*^2^ = 0.04) and group (*F* (2,109) = 4.87, *p* = 0.009, partial *η*^2^ = 0.082) effects were significant. Pairwise comparison after Bonferroni correction showed that the over-reporting group (*M* = 155.29, S.D. = 22.11) had higher Self-Centered Impulsivity score than the controls (*M* = 141.83, S.D. = 20.00, *p* = 0.029, *d* = 0.64) and the under-reporting group (*M* = 136.07, S.D. = 22.57, *p* = 0.011, *d* = 0.86), whereas the latter two groups did not differ significantly (*p* = 0.872).

**Figure 2:**
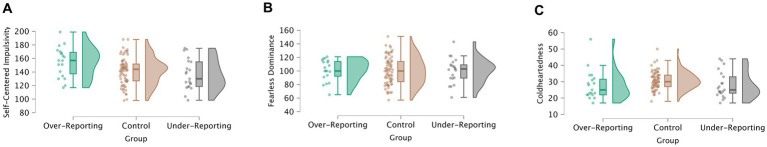
Distribution of the psychopathic dimension scores for three groups. **(A)** The over-reporting group scored higher on Self-Centered Impulsivity than the other two groups. **(B)** Groups did not differ on Fearless Dominance. **(C)** Groups did not differ on Coldheartedness.

When predicting Coldheartedness score, the model was significant, *F*(3,109) = 4.53, *p* = 0.005, partial *η*^2^ = 0.111. However, it was driven by the effect of gender, *F*(1,109) = 8.41, *p* = 0.005, partial *η*^2^ = 0.072. The effect of group was not significant, *F*(2,109) = 2.28, *p* = 0.107, partial *η*^2^ = 0.040. Similarly, when predicting Fearless Dominance score, the model was significant, *F*(3,109) = 3.04, *p* = 0.032, partial *η*^2^ = 0.077. However, it was driven by the effect of gender, *F*(1,109) = 9.03, *p* = 0.003, partial *η*^2^ = 0.076, but not group, *F*(2,109) = 0.09, *p* = 0.913, partial *η*^2^ = 0.002. Group differences are illustrated in Figures 2A–C.

## 4 Discussion

The present study examined how psychopathic traits relate to interoceptive processing during an ecologically valid social stressor. Two main findings emerged. First, interoceptive sensibility showed a clear and divergent pattern across psychopathy dimensions: Self-Centered Impulsivity and Coldheartedness were positively and negatively associated, respectively, with self-reported bodily sensations. Fearless Dominance showed no association with sensibility. Second, interoceptive accuracy—indexed by the match between physiological reactivity and subjective experience—was selectively related to Self-Centered Impulsivity. Individuals in the over-reporting group scored significantly higher on Self-Centered Impulsivity than both under-reporting and control participants, and these traits were negatively associated with mismatch scores when examined continuously. Neither Fearless Dominance nor Coldheartedness were associated with interoceptive accuracy. Taken together, these findings partially support our hypotheses: Self-Centered Impulsivity was strongly linked to over-reporting and heightened bodily sensibility as predicted, whereas Fearless Dominance did not show expected associations with under-reporting.

Theoretically, these findings refine current models of psychopathy by demonstrating that interoceptive disturbances differ meaningfully across psychopathy dimensions rather than reflecting a global deficit. Self-Centered Impulsivity, which captures emotional dysregulation, impulsivity, and stress reactivity, appears most consistently tied to interoceptive distortion. Elevated Self-Centered Impulsivity traits were associated with both heightened bodily sensibility and exaggerated subjective responding relative to physiological signals, consistent with frameworks describing this dimension as hyper-reactive, affectively labile, and prone to maladaptive stress responses (Edens and McDermott, 2010; Lilienfeld and Widows, 2005). In contrast, Fearless Dominance—often associated with emotional resilience, low anxiety, and attenuated stress responses—was not significantly associated with interoceptive measures. This suggests that low emotional reactivity in Fearless Dominance may not arise from under-perception of bodily signals but instead from upstream differences in affect generation or appraisal. Notably, this finding contrasts with Gao et al. (2012), who reported an association between the interpersonal-affective factor and mismatch scores. This discrepancy may reflect differences in sample characteristics and tasks across the two studies. Taken together, these findings align with emerging evidence that psychopathy is heterogeneous with regard to emotional and physiological processing (e.g., Dindo and Fowles, 2011; Patrick, 2022; Thomson et al., 2019), and they underscore that different psychopathy dimensions should not be expected to produce uniform interoceptive profiles.

Coldheartedness deserves particular attention. Conceptually akin to the callous affective traits measured in other psychopathy frameworks (e.g., PCL-R Facet 1, Triarchic Meanness, and callous-unemotional traits), Coldheartedness within the PPI-R has been criticized for its narrower content and weaker structural fit, and was therefore less studied (Benning et al., 2003; Berg et al., 2015). In the current study, Coldheartedness was unrelated to interoceptive accuracy but was significantly and negatively associated with interoceptive sensibility, suggesting that individuals higher on these traits may subjectively attend less to internal cues. This supports the notion that affective deficits in callous traits may reflect reduced engagement with emotional or bodily signals rather than impaired detection per se. However, because the Coldheartedness subscale has known psychometric limitations—restricted item content, low associations with externalizing behavior, and weaker alignment with broader affective psychopathy constructs (Berg et al., 2015)—its null associations with interoceptive accuracy should be interpreted cautiously. Future research may benefit from including additional measures of affective psychopathy (e.g., Inventory of Callous-Unemotional traits, Triarchic Meanness) to triangulate these effects.

Gender differences in psychopathic traits observed here replicate a robust pattern in the literature. Consistent with prior systematic reviews, men scored higher than women on all three dimensions of psychopathic traits, suggesting that the structure of psychopathy is relatively stable across gender while mean levels differ (Efferson and Glenn, 2018). Notably, gender did not interact with interoceptive variables, indicating that although men scored higher on psychopathic traits, the relationships between psychopathic traits and interoception were not gender specific. This reinforces the view that interoceptive correlates of psychopathy may operate similarly across men and women in community samples (Campos et al., 2023; Lamoureux and Glenn, 2021).

The inclusion of an ecologically valid Cyberball social exclusion paradigm provides important methodological insights. Social exclusion reliably induces stress and autonomic activation (Iffland et al., 2014; Kouchaki and Wareham, 2015; Lambe et al., 2019; Seeger et al., 2023), making it well suited for capturing real-time interoceptive processes. However, Cyberball task elicits primarily social-evaluative threat rather than fear or reward based decision-making. It is possible that differences related to Fearless Dominance would emerge more strongly under conditions involving physical threat, punishment anticipation, or high-stakes decision-making—contexts in which boldness typically manifests (Esteller et al., 2016; Yancey et al., 2019). Therefore, studies employing fear conditioning, aversive learning, or complex decision-making tasks may reveal complementary patterns and clarify the conditions under which interoceptive abnormalities appear across psychopathy dimensions.

Our findings also highlight the limitations of commonly used measures of interoceptive accuracy. The mismatch approach used here integrates physiological data with self-report during stress, offering greater ecological relevance than heartbeat detection tasks, which are prone to measurement error, expectancy biases, and conflation with beliefs about heart rate (See review by Desmedt et al., 2023; Ferentzi et al., 2025). The present pattern—Self-Centered Impulsivity associated with over-reporting and reduced accuracy—suggests that contextualized, task-based interoceptive metrics may better capture psychopathy-related abnormalities than decontextualized laboratory tasks. Broader adoption of dynamic accuracy indices may therefore advance understanding of interoception in antisocial/psychopathic behavior and improve predictive utility for real-world outcomes (Desmedt et al., 2023).

Finally, these results carry potential implications for recidivism reduction and intervention. Elevated Self-Centered Impulsivity, paired with distorted interoception, may contribute to impulsive decision-making, poor threat assessment, and maladaptive responses to interpersonal stress—processes linked to criminal behavior and reoffending. Interventions targeting interoceptive awareness, such as mindfulness-based programs, may prove especially beneficial for individuals high in these traits by helping calibrate bodily awareness and reducing emotional volatility. Conversely, individuals high in Fearless Dominance or Coldheartedness may require approaches targeting cognitive-affective processing rather than bodily awareness. For example, studies have suggested that transcranial direct current stimulation (tDCS) is effective in modulating response inhibition in participants high in Coldheartedness (Weidacker et al., 2016). These distinctions underscore the importance of tailoring intervention strategies to psychopathy profiles rather than assuming a one-size-fits-all model.

Several limitations warrant consideration. The sample was community-based and comprised of predominantly young adults, reducing generalizability to forensic populations in which psychopathy severity is typically higher and emotional deficits more pronounced. The Coldheartedness subscale’s limited construct breadth may have constrained detection of affective associations. The Cyberball paradigm, although ecologically valid, engages a specific type of stress that may not generalize to other emotional contexts. Finally, the current study focused solely on skin conductance as a measure of physiological activity; incorporating multi-system indices (e.g., cardiac, respiratory, neuroimaging) would provide a more comprehensive picture of interoceptive processes.

In conclusion, the present study demonstrates that psychopathic traits are differentially associated with interoceptive accuracy and sensibility in a social stress context. Self-Centered Impulsivity is characterized by heightened subjective bodily awareness and greater mismatch between perceived and physiological states, whereas Fearless Dominance and Coldheartedness show more limited associations. These findings contribute to a more nuanced understanding of emotional and interoceptive functioning in psychopathy and highlight the importance of multidimensional and ecologically grounded approaches to studying these relationships.

## Data Availability

The raw data supporting the conclusions of this article will be made available by the authors, without undue reservation.

## References

[ref1] Adolfi F. Couto B. Richter F. Decety J. Lopez J. Sigman M. . (2017). Convergence of interoception, emotion, and social cognition: a twofold fMRI meta-analysis and lesion approach. Cortex 88, 124–142. doi: 10.1016/j.cortex.2016.12.019, 28088652

[ref2] Babiak P. Neumann C. S. Hare R. D. (2010). Corporate psychopathy: talking the walk. Behav. Sci. Law 28, 174–193. doi: 10.1002/bsl.925, 20422644

[ref3] Benning S. D. Patrick C. J. Hicks B. M. Blonigen D. M. Krueger R. F. (2003). Factor structure of the psychopathic personality inventory: validity and implications for clinical assessment. Psychol. Assess. 15, 340–350. doi: 10.1037/1040-3590.15.3.340, 14593834

[ref4] Berardino S. D. Meloy J. R. Sherman M. Jacobs D. (2005). Validation of the psychopathic personality inventory on a female inmate sample. Behav. Sci. Law 23, 819–836. doi: 10.1002/bsl.666, 16333814

[ref5] Berg J. M. Hecht L. K. Latzman R. D. Lilienfeld S. O. (2015). Examining the correlates of the coldheartedness factor of the psychopathic personality inventory–revised. Psychol. Assess. 27, 1494–1499. doi: 10.1037/pas0000129, 25915788

[ref6] Cabrera A. Kolacz J. Pailhez G. Bulbena-Cabre A. Bulbena A. Porges S. W. (2018). Assessing body awareness and autonomic reactivity: factor structure and psychometric properties of the body perception questionnaire-short form (BPQ-SF). Int. J. Methods Psychiatr. Res. 27:e1596. doi: 10.1002/mpr.1596, 29193423 PMC6877116

[ref7] Campos C. Rocha N. B. Barbosa F. (2023). Dissociating cognitive and affective empathy across psychopathy dimensions: the role of interoception and alexithymia. Front. Psychol. 14:1082965. doi: 10.3389/fpsyg.2023.1082965, 37457066 PMC10345207

[ref8] Carré J. M. Hyde L. W. Neumann C. S. Viding E. Hariri A. R. (2013). The neural signatures of distinct psychopathic traits. Soc. Neurosci. 8, 122–135. doi: 10.1080/17470919.2012.703623, 22775289 PMC4709124

[ref9] Cleckley H. C. (1941). The mask of sanity. 1st Edn. St. Louis, MO: Mosby.

[ref10] Cohen J. (1988). Statistical power analysis for the behavioral sciences. 2nd Edn. Hillsdale, NJ: Lawrence Erlbaum.

[ref9001] Contreras-Rodríguez O. Pujol J. Batalla I. Harrison B. J. Bosque J. Ibern-Regas I. . (2014). Disrupted neural processing of emotional faces in psychopathy. Social cognitive and affective neuroscience, 9, 505–512. 23386739 10.1093/scan/nst014 PMC3989133

[ref11] Craig A. D. (2009). How do you feel--now? The anterior insula and human awareness. Nat. Rev. Neurosci. 10, 59–70. doi: 10.1038/nrn2555, 19096369

[ref12] Damasio A. R. (1994). Descartes' error: emotion, reason, and the human brain. New York: Grosset/Putnam.

[ref9003] Decety J. Skelly L. Yoder K. J. Kiehl K. A. (2014). Neural processing of dynamic emotional facial expressions in psychopaths. Social neuroscience, 9, 36–49. 24359488 10.1080/17470919.2013.866905 PMC3970241

[ref13] Desmedt O. Luminet O. Maurage P. Corneille O. (2025). Discrepancies in the definition and measurement of human interoception: a comprehensive discussion and suggested ways forward. Perspect. Psychol. Sci. 20, 76–98. doi: 10.1177/17456916231191537, 37642084

[ref14] Desmedt O. Luminet O. Walentynowicz M. Corneille O. (2023). The new measures of interoceptive accuracy: a systematic review and assessment. Neurosci. Biobehav. Rev. 153:105388. doi: 10.1016/j.neubiorev.2023.105388, 37708919

[ref15] Dindo L. Fowles D. (2011). Dual temperamental risk factors for psychopathic personality: evidence from self-report and skin conductance. J. Pers. Soc. Psychol. 100, 557–566. doi: 10.1037/a0021848, 21186933

[ref16] Drislane L. E. Patrick C. J. Sourander A. Sillanmäki L. Aggen S. H. Elonheimo H. . (2014). Distinct variants of extreme psychopathic individuals in society at large: evidence from a population-based sample. Personal. Disord. 5:154. doi: 10.1037/per0000060 24512459 PMC4091815

[ref17] Edens J. F. McDermott B. E. (2010). Examining the construct validity of the psychopathic personality inventory-revised: preferential correlates of fearless dominance and self-centered impulsivity. Psychol. Assess. 22, 32–42. doi: 10.1037/a0018220, 20230149

[ref18] Efferson L. M. Glenn A. L. (2018). Examining gender differences in the correlates of psychopathy: a systematic review of emotional, cognitive, and morality-related constructs. Aggress. Violent Behav. 41, 48–61. doi: 10.1016/j.avb.2018.05.009

[ref19] Esteller À. Poy R. Moltó J. (2016). Deficient aversive-potentiated startle and the triarchic model of psychopathy: the role of boldness. Biol. Psychol. 117, 131–140. doi: 10.1016/j.biopsycho.2016.03.012, 27033014

[ref20] Faul F. Erdfelder E. Lang A. G. Buchner A. (2007). G*power 3: a flexible statistical power analysis program for the social, behavioral, and biomedical sciences. Behav. Res. Methods 39, 175–191. doi: 10.3758/BF03193146, 17695343

[ref21] Ferentzi E. Vig L. Körmendi J. Witthöft M. Gerlach A. L. Pohl A. (2025). Cardiac interoceptive accuracy: an empirical comparison of three ability measures. Psychophysiology 62:e70078. doi: 10.1111/psyp.70078, 40468631 PMC12138236

[ref22] Gao Y. Raine A. Schug R. A. (2012). "Somatic aphasia": mismatch of body sensations with autonomic stress reactivity in psychopathy. Biol. Psychol. 90, 228–233. doi: 10.1016/j.biopsycho.2012.03.015, 22490763 PMC3372641

[ref23] Garfinkel S. N. Seth A. K. Barrett A. B. Suzuki K. Critchley H. D. (2015). Knowing your own heart: distinguishing interoceptive accuracy from interoceptive awareness. Biol. Psychol. 104, 65–74. doi: 10.1016/j.biopsycho.2014.11.004, 25451381

[ref9002] Gordon H. L. Baird A. A. End A. (2004). Functional differences among those high and low on a trait measure of psychopathy. Biological psychiatry, 56, 516–521. 15450788 10.1016/j.biopsych.2004.06.030

[ref24] Gunschera L. J. Brazil I. A. Driessen J. M. (2022). Social economic decision-making and psychopathy: a systematic review and meta-analysis. Neurosci. Biobehav. Rev. 143:104966. doi: 10.1016/j.neubiorev.2022.104966, 36403791

[ref25] Hare R. D. (2003). The hare psychopathy checklist-revised (PCL-R). 2nd Edn. Toronto: Multi-Health Systems.

[ref26] Huchzermeier C. Geiger F. Köhler D. Bruß E. Godt N. Hinrichs G. . (2008). Are there age-related effects in antisocial personality disorders and psychopathy? J. Forensic Leg. Med. 15, 213–218. doi: 10.1016/j.jflm.2007.10.002, 18423352

[ref27] Iffland B. Sansen L. M. Catani C. Neuner F. (2014). The trauma of peer abuse: effects of relational peer victimization and social anxiety disorder on physiological and affective reactions to social exclusion. Front. Psych. 5:26. doi: 10.3389/fpsyt.2014.00026, 24672491 PMC3957367

[ref28] Kofler L. (2023). You hurt my feelings: autonomic and behavioral responses to social exclusion and the moderating effect of psychopathic traits. City University of New York, New York: Doctoral dissertation.

[ref29] Kouchaki M. Wareham J. (2015). Excluded and behaving unethically: social exclusion, physiological responses, and unethical behavior. J. Appl. Psychol. 100, 547–556. doi: 10.1037/a0038034, 25314369

[ref30] Lambe L. J. Craig W. M. Hollenstein T. (2019). Blunted physiological stress reactivity among youth with a history of bullying and victimization: links to depressive symptoms. J. Abnorm. Child Psychol. 47, 1981–1993. doi: 10.1007/s10802-019-00565-y, 31111381

[ref31] Lamoureux V. A. Glenn A. L. (2021). An analysis of conscious fear and automatic threat response in psychopathy. Personal. Disord. Theory Res. Treat. 12, 171–181. doi: 10.1037/per0000406, 32658520

[ref33] Lilienfeld S. O. Widows M. R. (2005). Psychopathic personality inventory-revised: professional manual. Lutz, FL: Psychological Assessment Resources Inc.

[ref32] Lilienfeld S. O. Latzman R. D. Watts A. L. Smith S. F. Dutton K. (2014). Correlates of psychopathic personality traits in everyday life: results from a large community survey. Front. Psychol. 5:740. doi: 10.3389/fpsyg.2014.00740, 25101019 PMC4106400

[ref34] Lykken D. T. (1995). The Antisocial Personalities (1st ed.). New York: Psychology Press. doi: 10.4324/9780203763551

[ref35] Lyons M. Hughes S. (2015). Feeling me, feeling you? Links between the dark triad and internal body awareness. Pers. Individ. Differ. 86, 308–311. doi: 10.1016/j.paid.2015.06.039

[ref36] Ma D. S. Correll J. Wittenbrink B. (2015). The Chicago face database: a free stimulus set of faces and norming data. Behav. Res. Methods 47, 1122–1135. doi: 10.3758/s13428-014-0532-5, 25582810

[ref9005] Marcus D. K. Fulton J. J. Edens J. F. (2013). The two-factor model of psychopathic personality: evidence from the psychopathic personality inventory. Personality Disorders: Theory, Research, and Treatment, 4:67. 10.1037/a0025282 23355982

[ref37] Maurer J. M. Edwards B. G. Harenski C. L. Decety J. Kiehl K. A. (2022). Do psychopathic traits vary with age among women? A cross-sectional investigation. J. Forensic Psychiatry Psychol. 33, 112–129. doi: 10.1080/14789949.2022.2036220, 35221799 PMC8865477

[ref38] Mehling W. E. Price C. Daubenmier J. J. Acree M. Bartmess E. Stewart A. (2012). The multidimensional assessment of interoceptive awareness (MAIA). PLoS One 7:e48230. doi: 10.1371/journal.pone.0048230, 23133619 PMC3486814

[ref9006] Miller J. D. Lynam D. R. (2012). An examination of the Psychopathic Personality Inventory’s nomological network: a meta-analytic review. Personality Disorders: Theory, research, and treatment, 3:305. 10.1037/a0024567 22452764

[ref39] Murphy J. Brewer R. Plans D. Khalsa S. S. Catmur C. Bird G. (2020). Testing the independence of self-reported interoceptive accuracy and attention. Q. J. Exp. Psychol. 73, 115–133. doi: 10.1177/1747021819879826, 31519137

[ref40] Murphy J. Catmur C. Bird G. (2019). Classifying individual differences in interoception: implications for the measurement of interoceptive awareness. Psychon. Bull. Rev. 26, 1467–1471. doi: 10.3758/s13423-019-01632-7, 31270764 PMC6797703

[ref41] Nentjes L. Meijer E. Bernstein D. Arntz A. Medendorp W. (2013). Brief communication: investigating the relationship between psychopathy and interoceptive awareness. J. Personal. Disord. 27, 617–624. doi: 10.1521/pedi_2013_27_105, 23786270

[ref42] Neumann C. S. Hare R. D. (2008). Psychopathic traits in a large community sample: links to violence, alcohol use, and intelligence. J. Consult. Clin. Psychol. 76, 893–899. doi: 10.1037/0022-006X.76.5.893, 18837606

[ref43] Patrick C. J. (2022). Psychopathy: current knowledge and future directions. Annu. Rev. Clin. Psychol. 18, 387–415. doi: 10.1146/annurev-clinpsy-072720-012851, 35119947

[ref44] Patrick C. J. Fowles D. C. Krueger R. F. (2009). Triarchic conceptualization of psychopathy: developmental origins of disinhibition, boldness, and meanness. Dev. Psychopathol. 21, 913–938. doi: 10.1017/S0954579409000492, 19583890

[ref45] Plans D. Ponzo S. Morelli D. Cairo M. Ring C. Keating C. T. . (2021). Measuring interoception: the phase adjustment task. Biol. Psychol. 165:108171. doi: 10.1016/j.biopsycho.2021.108171, 34411620

[ref46] Puzzanchera C. M. Hockenberry S. Sickmund M. (2022). Youth and the juvenile justice system: 2022 national report. Pittsburgh, PA: National Center for Juvenile Justice.

[ref47] Schandry R. (1981). Heart beat perception and emotional experience. Psychophysiology 18, 483–488. doi: 10.1111/j.1469-8986.1981.tb02486.x, 7267933

[ref9004] Seara-Cardoso A. Sebastian C. L. Viding E. Roiser J. P. (2016). Affective resonance in response to others’ emotional faces varies with affective ratings and psychopathic traits in amygdala and anterior insula. Social Neuroscience, 11, 140–152. 25978492 10.1080/17470919.2015.1044672 PMC5321475

[ref48] Seeger N. A. Brackmann N. Lamm C. Hennig-Fast K. Pfabigan D. M. (2023). Social exclusion evokes different psychophysiological responses in individuals high on the psychopathy facets fearless dominance and self-centered impulsivity. Front. Psych. 14:1197595. doi: 10.3389/fpsyt.2023.1197595, 38274437 PMC10808528

[ref49] Skeem J. L. Cauffman E. (2003). Views of the downward extension: comparing the youth version of the psychopathy checklist with the youth psychopathic traits inventory. Behav. Sci. Law 21, 737–770. doi: 10.1002/bsl.563, 14696029

[ref50] Thomson N. D. Aboutanos M. Kiehl K. A. Neumann C. Galusha C. Fanti K. A. (2019). Physiological reactivity in response to a fear-induced virtual reality experience: associations with psychopathic traits. Psychophysiology 56:e13276. doi: 10.1111/psyp.13276, 30129671

[ref51] Weidacker K. Weidemann C. T. Boy F. Johnston S. J. (2016). Cathodal tDCS improves task performance in participants high in Coldheartedness. Clin. Neurophysiol. 127, 3102–3109. doi: 10.1016/j.clinph.2016.05.274, 27472546

[ref52] Williams K. D. (2009). Ostracism: a temporal need-threat model. Adv. Exp. Soc. Psychol. 41, 275–314. doi: 10.1016/S0065-2601(08)00406-1

[ref54] Williams K. D. Jarvis B. (2006). Cyberball: a program for use in research on interpersonal ostracism and acceptance. Behav. Res. Methods 38, 174–180. doi: 10.3758/BF03192765, 16817529

[ref53] Williams K. D. Cheung C. K. Choi W. (2000). Cyberostracism: effects of being ignored over the internet. J. Pers. Soc. Psychol. 79, 748–762. doi: 10.1037/0022-3514.79.5.748, 11079239

[ref55] Yancey J. R. Bowyer C. B. Foell J. Boot W. R. Patrick C. J. (2019). Boldness moderates the effects of external threat on performance within a task-switching paradigm. J. Exp. Psychol. Hum. Percept. Perform. 45, 758–770. doi: 10.1037/xhp0000631, 30945907

[ref56] Yang Y. Raine A. (2009). Prefrontal structural and functional brain imaging findings in antisocial, violent, and psychopathic individuals: a meta-analysis. Psychiatry Res. Neuroimaging 174, 81–88. doi: 10.1016/j.pscychresns.2009.03.012, 19833485 PMC2784035

[ref57] Zwets A. J. Hornsveld R. Kraaimaat F. W. Kanters T. Muris P. van Marle H. (2014). The psychometric properties of the anger bodily sensations questionnaire (ABSQ). J. Forensic Psychiatry Psychol. 25, 432–450. doi: 10.1080/14789949.2014.925957

